# Attenuation of traumatic brain injury-induced cognitive impairment in mice by targeting increased cytokine levels with a small molecule experimental therapeutic

**DOI:** 10.1186/s12974-015-0289-5

**Published:** 2015-04-10

**Authors:** Adam D Bachstetter, Scott J Webster, Danielle S Goulding, Jonathan E Morton, D Martin Watterson, Linda J Van Eldik

**Affiliations:** Sanders-Brown Center on Aging, University of Kentucky, 800 S Limestone Street, Lexington, KY USA; Department of Pharmacology, Northwestern University, 303 E Chicago Avenue, Chicago, IL USA; Department of Anatomy and Neurobiology, University of Kentucky, 800 Rose Street, Lexington, KY USA

**Keywords:** Cytokines, Glia, Interleukin, Neuroinflammation, Drug discovery, Microglia, Astrocytes, Traumatic brain injury, Closed head injury, Cognitive dysfunction

## Abstract

**Background:**

Evidence from clinical studies and preclinical animal models suggests that proinflammatory cytokine overproduction is a potential driving force for pathology progression in traumatic brain injury (TBI). This raises the possibility that selective targeting of the overactive cytokine response, a component of the neuroinflammation that contributes to neuronal dysfunction, may be a useful therapeutic approach. MW151 is a CNS-penetrant, small molecule experimental therapeutic that selectively restores injury- or disease-induced overproduction of proinflammatory cytokines towards homeostasis. We previously reported that MW151 administered post-injury (p.i.) is efficacious in a closed head injury (CHI) model of diffuse TBI in mice. Here we test dose dependence of MW151 to suppress the target mechanism (proinflammatory cytokine up-regulation), and explore the therapeutic window for MW151 efficacy.

**Methods:**

We examined suppression of the acute cytokine surge when MW151 was administered at different times post-injury and the dose-dependence of cytokine suppression. We also tested a more prolonged treatment with MW151 over the first 7 days post-injury and measured the effects on cognitive impairment and glial activation.

**Results:**

MW151 administered up to 6 h post-injury suppressed the acute cytokine surge, in a dose-dependent manner. Administration of MW151 over the first 7 days post-injury rescues the CHI-induced cognitive impairment and reduces glial activation in the focus area of the CHI.

**Conclusions:**

Our results identify a clinically relevant time window post-CHI during which MW151 effectively restores cytokine production back towards normal, with a resultant attenuation of downstream cognitive impairment.

## Introduction

Traumatic brain injury (TBI) is a public health problem associated with injuries sustained by military personnel, concussion-related sports injuries, and falls in the elderly [1]. TBI initiates a cascade of complex pathophysiological events that can themselves add to physiological injury and the longer-term morbidity related to neurodegenerative complications. For example, TBI increases susceptibility for development of Alzheimer’s disease (AD) and other dementias, post-traumatic epilepsy, neuropsychiatric disorders, post-traumatic stress disorder, and chronic traumatic encephalopathy. The diverse neurologic sequelae of TBI can occur months, years, even decades after the last head injury [2-4]. Thus, it is critical to elucidate the underlying pathophysiology by which an initial TBI elicits an enhanced susceptibility to subsequent injuries and neurologic complications.

A key mechanism that drives pathophysiology progression in many CNS disorders involves abnormal inflammatory responses in the brain, termed neuroinflammation (for recent reviews, see [5,6]). Studies with animal models and human head injury patients suggest that proinflammatory cytokine overproduction is one component of the neuroinflammation that contributes to adverse outcomes following TBI [7,8]. This raises the importance of exploring attenuation of proinflammatory cytokine overproduction as a therapeutic target.

We developed [9] a CNS-penetrant, small molecule experimental therapeutic, MW01-2-151WH (MW151), that selectively restores injury- or disease-induced overproduction of proinflammatory cytokines towards homeostasis. MW151 is efficacious in a variety of animal models of diseases where proinflammatory cytokine overproduction is a component of disease progression, including (1) animal models of Alzheimer’s disease [9,10]; (2) neurologic sequelae of seizures [11,12]; (3) EAE model of multiple sclerosis [13]; (4) a ‘two-hit’ injury model of increased susceptibility to seizures after a TBI [14], and (5) radiation-induced cognitive impairment [15]. Particularly relevant to the current study, we have previously reported [16] that MW151 is efficacious in a closed head injury (CHI) model of diffuse TBI in mice, when administered during the time of the acute post-injury cytokine surge. The observation that MW151 attenuates disease- and injury-induced proinflammatory cytokine overproduction and reduces downstream synaptic and cognitive dysfunction in multiple animal models of CNS disorders suggests that further development of MW151 is warranted.

A key component of developing proinflammatory cytokine modulators for altering disease progression is consideration of dosing. Dosing is the pharmacological basis of therapeutic action and includes the amount of drug administered, the frequency of administration, and the time window during disease progression for administration. The goal of this study was to probe the time window and concentration dependence of MW151 intervention to suppress the targeted mechanism, proinflammatory cytokine up-regulation, and the impact of appropriate dosing on efficacy and attenuation of downstream cognitive dysfunction.

## Materials and methods

### Animals

The Institutional Animal Care and Use Committee of the University of Kentucky approved the use of animals in this study, which were conducted in accordance with the principles of animal care and experimentation in the Guide for the Care and Use of Laboratory Animals. All experiments used adult male C57BL/6 J mice obtained from Jackson Laboratory. Animal experiments followed the recent NIH guidelines for rigor in study design and analysis [17,18], including randomization of animals and blinding of treatment groups and tissue samples.

### Surgical procedure

Adult mice were subjected to a CHI using a stereotaxic electromagnetic impactor [19]. Under continuous inhalation of isoflurane (3.5%, 1 liter/min) anesthesia, with the head stabilized using ear bars in a digital mouse stereotaxic frame (Stoelting Co., Wood Dale, USA), a midline sagittal scalp incision was made. To displace the force of the impact, a 1-ml latex pipette bulb (ThermoFisher Scientific, Waltham, USA) was placed under the head and filled with water. A 5.0-mm steel tip impounder (Leica Biosytems, Wetzlar, Germany) was used to deliver a single controlled midline cortical impact, delivered at coordinates: ML = 0.0 mm; AP = −1.5 mm, with a controlled velocity (5.0 ± 0.2 m/s), dwell time (100 ms), and impact depth (1.0 mm). Mice with depressed skull fracture or visible hemorrhage were excluded from the study. Sham-injured mice underwent identical surgical procedures as the trauma group, but no impact was delivered. The time elapsed until the animal spontaneously rights was recorded as an acute neurological assessment, and defined as the righting reflex time. In this mild diffuse injury model, the mortality rate is <1%. Following each surgery, animals were monitored continuously until they have fully recovered.

### Synthesis and use of MW151

MW01-2-151SRM (2-(4-(4-methyl-6-phenylpyridazin-3-yl)piperazin-1-yl)pyrimidine) was synthesized and characterized as previously reported [9]. As previously described [10], MW151 was dissolved in 0.9% sterile NaCl (saline: Hospira NDC 0409-4888-10), and administered by intraperitoneal (i.p.) injection. Saline was injected i.p. as the vehicle control.

### Radial arm water maze behavior

A 2-day radial arm water maze (RAWM) protocol was used as previously described [20]. Briefly, in block 1 (first 6 trials) and block 2 (6 trials), mice were trained to identify the platform location by alternating between a visible and a hidden platform (3 hidden platform trials and 3 visible platform trials for each block). Block 3 (3 trials) used only a hidden platform. The next day, mice were tested in 3 blocks of 5 trials each (blocks 4 to 6; 15 total trials), all using only a hidden platform to test their spatial memory retention. EthoVision XT 8.0 video tracking software (Noldus Information Technology, Wageningen, The Netherlands) was used to record and score the behavior. Data are presented as the average errors per block during the hidden platform trials.

### Brain tissue harvesting, biochemical, and immunohistochemical endpoints

Mice were deeply anesthetized with 5% isoflurane prior to transcardial perfusion with ice-cold PBS for 5 min, then the brains were rapidly removed and dissected, as previously described [10,21]. Cytokine and chemokine levels were measured in brain homogenates using Meso Scale Discovery (MSD) ELISA, as previously described [10,21]. Immunohistochemical (IHC) staining was done following established methods, and quantified using the Aperio ScanScope XT digital slidescanner and Aperio ImageScope software positive pixel count algorithm (version 9), as previously described [10,21]. Primary antibodies used included rabbit anti-glial fibrillary acidic protein (GFAP) (Dako Cat#Z0334; (1:10,000); Dako, Glostrup, Denmark); rabbit anti-IBA1 (Wako Cat#019-19741; (1:10,000); Wako Chemicals USA Inc., Richmond, USA); and rat anti-CD45 (YW62.3) (ThermoFisher Scientific Cat#MA1447081; (1:10,000); ThermoFisher Scientific, Waltham, USA).

### Statistics

JMP software version 10.0 was used for statistical analysis. A repeated measures ANOVA was used for RAWM. For all other endpoints, a one-way ANOVA was used to examine differences within those factors. A two-tailed Student’s *t* test was used for *post hoc* analysis to compare only the effect of injury compared to sham and to compare the effect of MW151 compared to vehicle in the injured mice, as these comparisons were determined *a priori* to be the ones of interest. Differences between means were considered significant at *α* = 0.05. Graphs were generated using GraphPad Prism software version 6.0, and values are expressed as mean ± SEM, unless otherwise noted.

## Results and discussion

### Selection of preclinical model and validation of mechanism of action

In the field of neurotrauma, a number of preclinical models have been characterized (for review of rodent TBI protocols, see [22]). Because no single animal model can reproduce the complexity of TBI pathology seen in humans, it is important to choose a model that has an injury with some relevance to the clinical paradigm. Most important for pharmacological studies, the model should exhibit changes in the targeted pathways in order to test mechanistic pharmacodynamics, or how drug intervention alters the mechanism of action. For this study, we chose a midline CHI protocol that uses an electromagnetic impactor and produces a diffuse brain injury without focal brain lesions, fracture of the skull, or hemorrhages. Mild diffuse injury after closed-skull impact is similar to the injury characteristics observed most commonly in human head injury. In addition, this experimental protocol produces a reproducible injury with low mortality, making it technically feasible for pharmacological studies.

MW151 was developed [9] using the classic functional approach with the focus on a deliverable with the following desired pharmacological properties: selective suppression of stressor-induced up-regulation of neuroinflammatory responses of activated glia such as proinflammatory cytokine overproduction. An advantage of the functional approach is the unbiased focus on attenuation of pathology progression, with potential for identification of alternative therapeutic strategies, which has resulted in historical success in delivering widely used drugs to clinical practice. Our discovery process to develop novel small molecules began with a validated mouse model [23-25] and discovery engine that used novel molecule design based on pharmacoinformatics and a curated database of CNS-penetrant drugs. The starting point for the family of compounds encompassing MW151 and its analogs was a chemical scaffold present in previous clinically useful CNS drugs. The strict exclusion criteria of the approach resulted in the synthesis and testing of fewer than two dozen novel small molecules to find viable hits. Focused medicinal chemistry refinement was then done to improve metabolic stability and pharmacokinetic features. MW151 was one of two best-in-class deliverables from the discovery engine’s hierarchal pharmacological screening process.

MW151 is a water-soluble, chemically stable, small molecule (423 MW) that is orally bioavailable and CNS-penetrant, with a brain:blood ratio >1, similar to other CNS drugs in clinical use or under development. MW151 is selective in its action and is not a pan-suppressor of neuroinflammation. For example, MW151 suppresses disease- and injury-induced overproduction of proinflammatory cytokines such as IL-1β and TNFα, but does not block anti-inflammatory cytokines such as IL-10 [10]. MW151’s pharmacological mechanism of action is one that restores activated pathways back towards homeostasis, as shown by its lack of effect in control animals and its failure to depress basal cytokine levels at efficacious doses. Further, efficacy is achieved in the absence of any signs of general immunosuppression or non-selective anti-inflammatory action [9-16,26]. Therefore, future development of MW151 could have broad clinical applications for a number of other CNS disorders where proinflammatory cytokine dysregulation is part of the pathophysiology progression mechanism and restoration towards homeostasis is the desired therapeutic goal.

**Figure 1:**
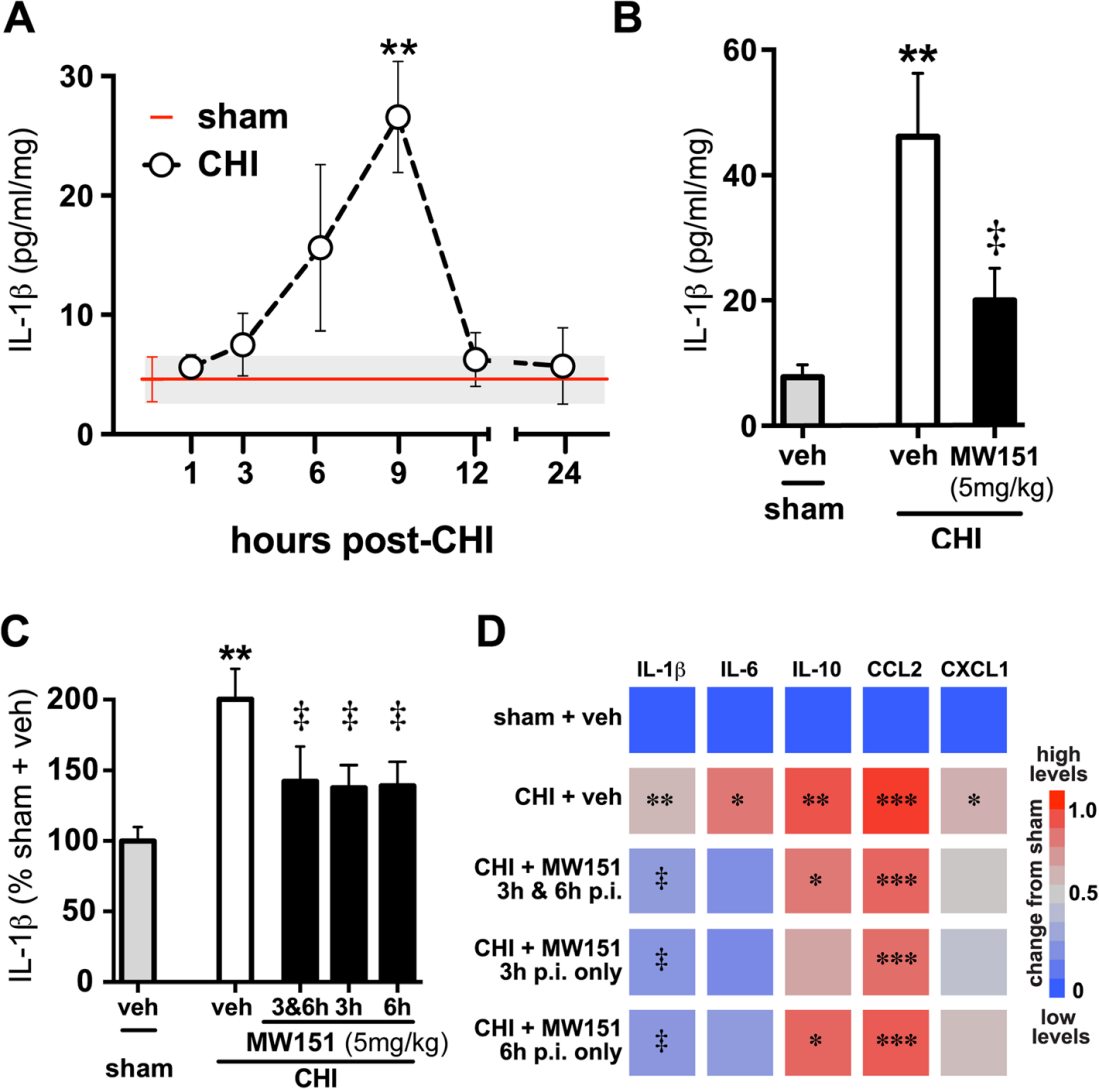
**Time window for suppression of acute cytokine surge by MW151. (A)** Temporal changes in IL-1β protein levels were measured in tissue homogenates from the cortex of adult C57BL/6 J mice subjected to sham conditions (red line mean ± SEM), or at select time points after the CHI (*n* = 4 per group). **(B)** Treatment of mice with MW151 (5 mg/kg i.p.) at 3 and 6 h post-injury (p.i.) significantly reduced IL-1β up-regulation at 9 h p.i., compared to CHI + saline vehicle (veh) control (*n* = 4 sham; *n* = 9 CHI + veh; *n* = 10 CHI + MW151). **(C, D)** Injured mice were treated with veh or MW151 (5 mg/kg i.p.) at 3 h and 6 h, at 3 h only, or at 6 h only, and cytokine and chemokine protein levels were measured in cortex homogenates at 9 h p.i. (*n* = 7 to 20 per group). **(C)** Injury-induced IL-1β levels were significantly suppressed with MW151 regardless of time of treatment. **(D)** Heatmap of the relative changes in cytokine and chemokine protein levels showing the specificity of MW151 for IL-1β and IL-6. Significance for a given inflammatory marker is indicated by *P < 0.05, **P < 0.005, ***P < 0.001 compared to sham treated mice. ‡*P* < 0.05 compared to CHI + veh.

A critical first step before testing MW151 in the CHI model was to validate that the model exhibits an injury-induced acute proinflammatory cytokine surge, the mechanism of action targeted by MW151. Therefore, we subjected mice to CHI and measured IL-1β levels in the cortex at various post-injury (p.i.) time points. As shown in Figure 1A, levels of IL-1β in the cortex began to rise at 3 h p.i., peaked at 9 h p.i., and returned to pre-injury levels by 12 to 24 h p.i. This pattern of the acute cytokine surge is similar to that found previously in a comparable CHI model [16], although that CHI protocol resulted in a slightly delayed peak IL-1β level (12 h). The reason for the slight temporal difference in IL-1β response is unknown, but could reflect strain differences in the mice (C57BL/6 J *vs*. CD-1), or minor variations in the CHI models. Previously, Lloyd *et al*. showed that administration of MW151 (5 mg/kg i.p.) at two post-injury time points when cytokines were increasing (3 h and 9 h) significantly reduced the peak (12 h p.i.) of the proinflammatory cytokine (that is, IL-1β) surge in the CNS [16]. To confirm that MW151 administration in our midline CHI model would suppress IL-1β, we injected MW151 (5 mg/kg i.p.) at two time points (3 h and 6 h) during the acute rise in IL-1β, and harvested brain tissue at the 9-h p.i. peak IL-1β time point. As shown in Figure 1B, MW151 administered under this treatment paradigm significantly reduced the IL-1β levels in the cortex at 9 h p.i., demonstrating engagement of the pharmacological mechanism of action by MW151.

### Intervention time window for suppression of acute cytokine surge by MW151

After confirming that our CHI model showed the predicted acute cytokine surge and that two administrations of MW151 suppressed this surge, we tested whether a single injection of MW151 administered at different times after injury would be effective at inhibiting IL-1β levels. We tested intervention at two clinically relevant time windows, 3 h or 6 h following CHI. MW151 (5 mg/kg i.p.) was injected at either 3 or 6 h p.i., brain harvested at the 9-h p.i. time point, and IL-1β levels in cortex homogenates determined. A positive control group that received two injections of MW151 (at 3 and 6 h p.i.) was also included. In the vehicle-treated groups (sham or CHI), there was no difference in IL-1β levels in the groups treated at 3, 6, or 3 and 6 h p.i.; therefore, these vehicle treatment groups were combined into a single sham + veh or CHI + veh group (Figure 1C,D; Table 1). Strikingly, we found that MW151 (5 mg/kg) significantly reduced IL-1β levels almost to pre-injury levels, not only when two injections were given (3 and 6 h), but even after a single administration at either 3 or 6 h p.i. (58%, 63%, and 61% decrease from CHI + veh, respectively) (Figure 1C). MW151 administration also reduced IL-6 levels (Figure 1D), but the decrease did not reach statistical significance. In addition, we found that MW151 did not significantly reduce the levels of the anti-inflammatory cytokine IL-10, or the chemokines CCL2 or CXCL1 (Figure 1D). The lack of effect of MW151 on CCL2 levels in our CHI model is in contrast to previous findings [16] in a different CHI model, but the reason for this difference was not explored further. Our data with acute administration of MW151 demonstrate that the injury-induced IL-1β levels are inhibited even when MW151 is administered at 6 h p.i. and that the compound shows selectivity for IL-1β and IL-6 compared to the other markers measured.

**Table 1:** **Levels of cytokines and chemokines in the cortex at the 9**-**h p.i. time point**

**Treatment group**	**IL-1β**	**IL-6**	**IL-10**	**CCL2**	**CXCL1**
sham + veh (*n* = 7)	4.9 ± 1.3	16.1 ± 10.8	1.3 ± 2.0	53.0 ± 7.6	103.8 ± 100.2
CHI + veh (*n* = 20)	9.8 ± 4.7	41.0 ± 39.1	7.2 ± 5.8	445.1 ± 240.4	274.0 ± 211.2
CHI + MW151 at 3 h and 6 h (*n* = 10)	7.0 ± 3.8	22.0 ± 13.7	6.5 ± 3.0	403.3 ± 134.5	235.6 ± 125.9
CHI + MW151 at 3 h (*n* = 10)	6.8 ± 2.5	21.4 ± 15.4	5.6 ± 4.6	393.5 ± 134.6	214.6 ± 132.1
CHI + MW151 at 6 h (*n* = 9)	6.8 ± 2.5	23.7 ± 21.7	6.9 ± 4.3	414.2 ± 134.5	246.9 ± 88.3

Data are expressed as pg/ml/mg (mean ± SD). CHI, closed head injury; veh, vehicle.

### Concentration dependence of MW151 inhibition of post-injury IL-1β up-regulation

**Figure 2:**
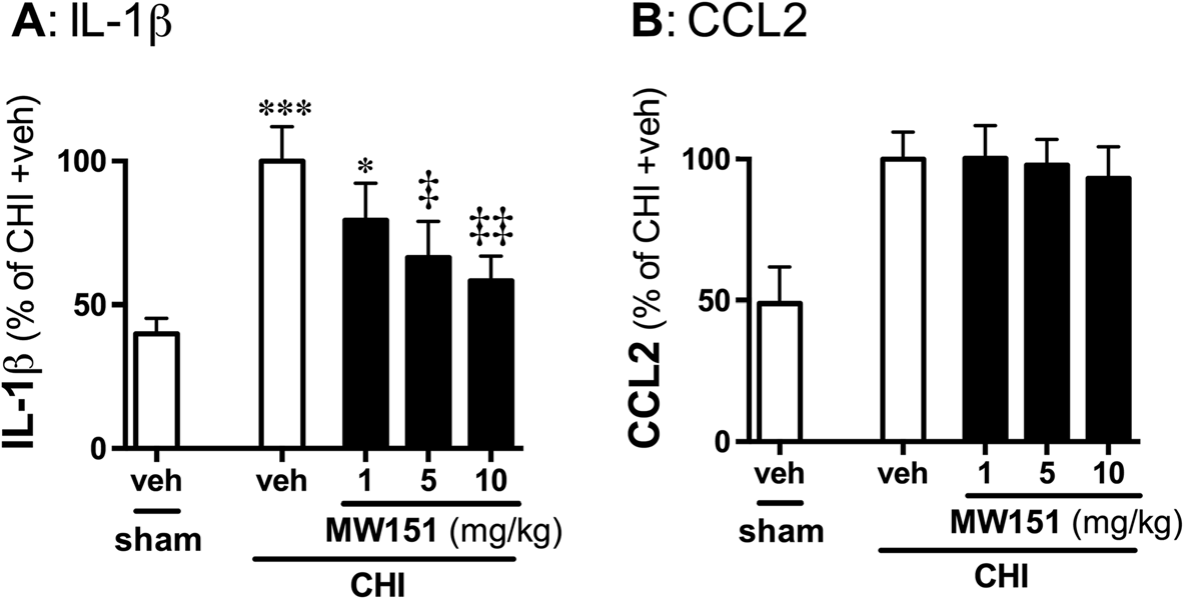
**Dose-dependent suppression of IL-1β up-regulation by MW151.** Mice were subjected to sham injury or CHI, and injected i.p. at 6 h p.i. with saline vehicle (veh). CHI mice were injected i.p. at 6 h p.i. with MW151 at one of three different doses (1, 5, and 10 mg/kg). Levels of IL-1β **(A)** and CCL2 **(B)** protein were measured in cortex homogenates at the 9-h p.i. time point. *P < 0.05, ***P < 0.001 compared to sham mice. ‡*P* < 0.05, ‡‡*P* < 0.01 CHI + veh compared to CHI + MW151 (*n* = 10 sham + veh; *n* = 20 CHI + veh; *n* = 9 to 10 CHI + MW151).

To determine whether MW151 showed dose-dependent suppression of IL-1β in the CNS following a CHI, we tested three different doses of MW151 (1, 5, and 10 mg/kg) administered at a single time (6 h p.i.). Brain tissue was harvested at 9 h p.i., and levels of IL-1β and CCL2 in the cortex were measured, to assess the target mechanism of inhibiting proinflammatory cytokine overproduction (IL-1β) and to assess the selectivity of MW151 by determining if it would suppress CCL2 levels at higher doses. As shown in Figure 2, MW151 showed dose-dependent suppression of IL-1β up-regulation, with significant inhibition at doses of 5 and 10 mg/kg. MW151 had no effect on injury-induced CCL2 levels at any dose tested.

### Functional efficacy of MW151 to suppress CHI-induced cognitive impairment

**Figure 3:**
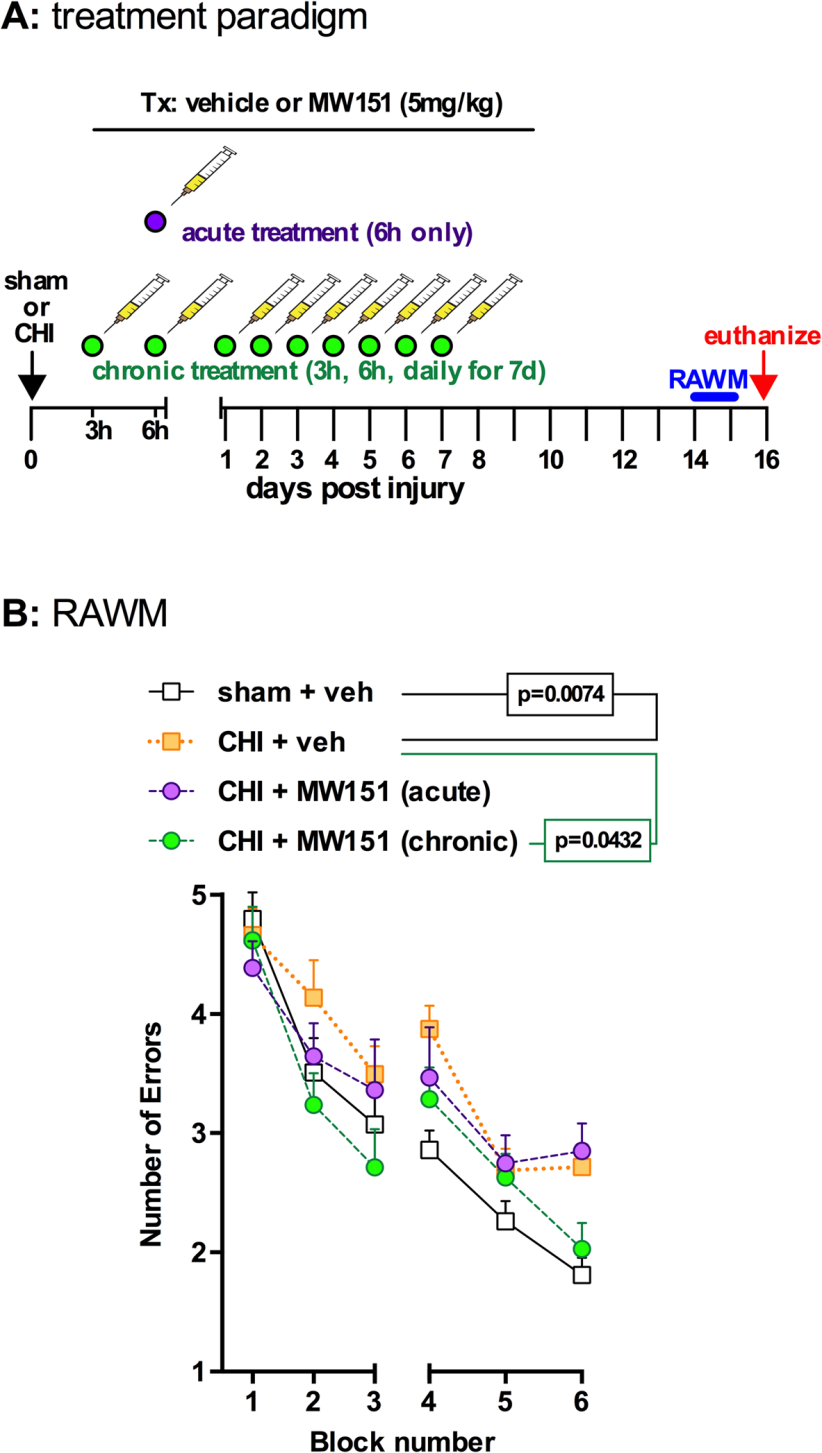
**Functional efficacy of MW151 to suppress CHI-induced cognitive impairment. (A)** Overview of study design. MW151 (5 mg/kg i.p.) was administered either as a single acute treatment at 6 h p.i. or in a more extended treatment paradigm (3 h, 6 h, then once daily for 7 days p.i.). **(B)** Mice were tested for cognitive deficits in the RAWM starting at 14 days p.i. (*n* = 23 sham; *n* = 27 CHI + veh; *n* = 12 CHI + MW150 acute; *n* = 14 CHI + MW150 chronic).

We tested the potential of MW151 to suppress injury-induced cognitive deficits in two different treatment paradigms (Figure 3A). In the first paradigm, MW151 (5 mg/kg) was administered at 3 and 6 h p.i., and then once daily for 7 days. This extended treatment approach uses a similar dosing paradigm to a previous report showing that a structural analog of MW151 can rescue TBI-induced cognitive impairments [27]. In the second paradigm, a single dose of MW151 (5 mg/kg) was given at the 6-h p.i. time point. In both treatment paradigms, mice were tested starting at day 14 p.i. for cognitive impairment in the RAWM. The rationale for this experimental design was to test if suppression of the peak IL-1β response with a single administration of MW151 given at 6 h p.i. would be sufficient to prevent subsequent cognitive impairment assessed 2 weeks later or if a more extended treatment was required.

As shown in Figure 3B, the CHI mice treated with vehicle showed a significant impairment in RAWM compared to the sham group. MW151 administered under the extended treatment paradigm (3 h, 6 h, and 1 to 7 days p.i.) led to a significant improvement in RAWM performance. However, a single injection of MW151 at 6 h p.i. did not reduce the CHI-induced cognitive impairment in the RAWM assessed 2 weeks later. There are several possible interpretations of these results. It is possible that a single 6-h p.i. administration of MW151, while effective at suppressing the peak of the IL-1β response, may be too late to inhibit other key proinflammatory cytokines that show an earlier post-injury surge and that may contribute to subsequent impairment. It is also possible that other detrimental neuroinflammatory responses that contribute to cognitive impairment occur during the first 7 days p.i. and that administration of MW151 during this time window inhibits those responses. Finally, it is possible that a single administration of MW151 is not sufficient to prevent cognitive deficits but that functional efficacy requires a more prolonged administration of drug. Future studies exploring the therapeutic efficacy window in more detail will be required to address these possibilities.

### Effects of MW151 on glia morphological changes

**Figure 4:**
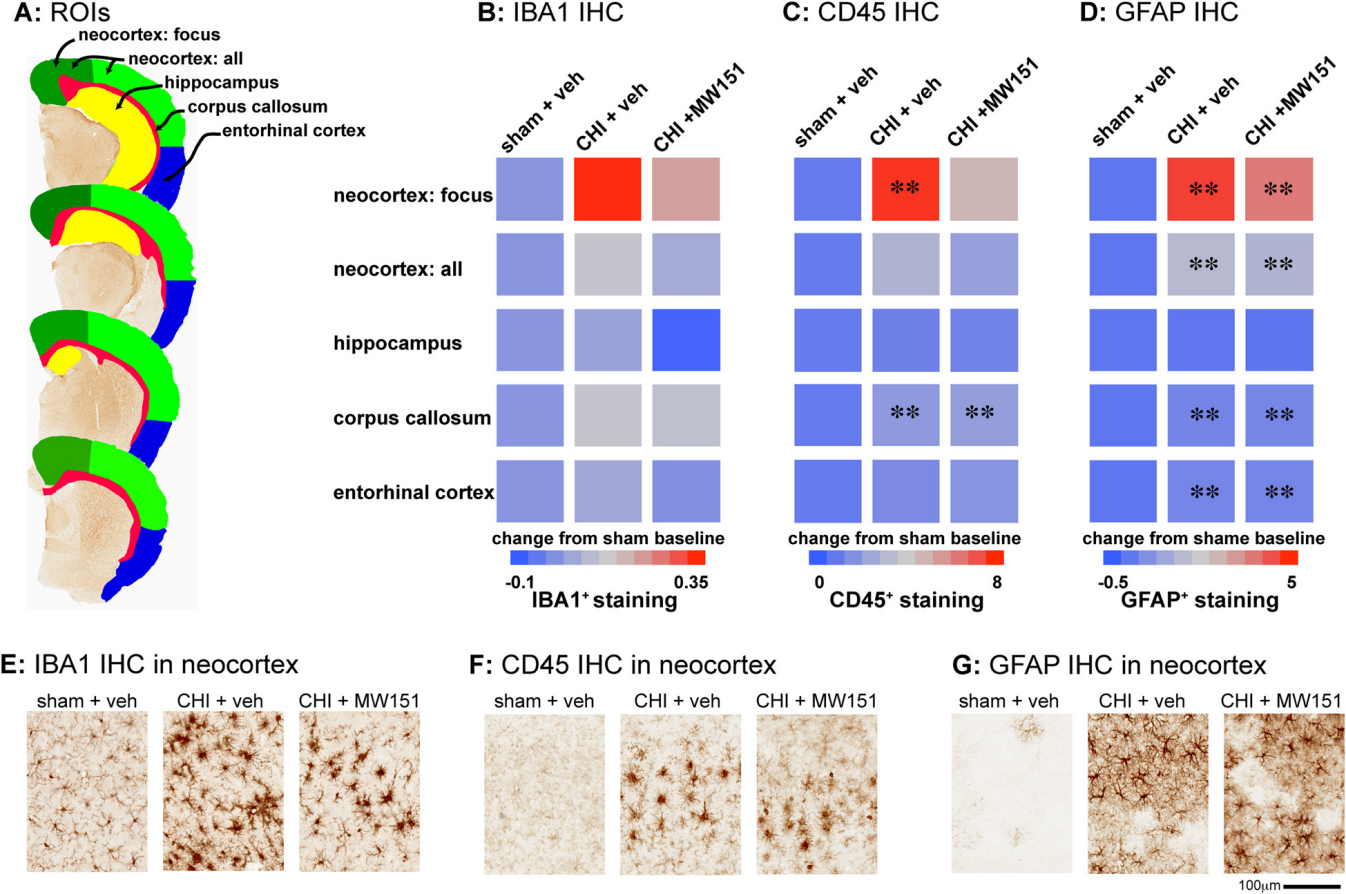
**Effects of MW151 treatment on glia morphological activation.** Mice were subjected to sham injury or CHI and administered veh control; injured mice were administered MW151 (5 mg/kg i.p.) at 3 h, 6 h, and once daily for 7 days p.i.. Brain tissue was prepared for IHC at 16 days p.i. and stained for microglia (IBA1, CD45) and astrocyte (GFAP) morphological activation. **(A)** Overview of regions of interest (ROIs) used for quantitative neuropathological analysis of glia activation using the Aperio ScanScope. Heatmap shows the effect of injury and MW151 treatment in five ROIs for IBA1 **(B)**, CD45 **(C)**, and GFAP **(D)** IHC. Treatment with MW151 reduced IBA1, CD45, and GFAP staining in the focus region of the cortex. There was little effect of MW151 treatment on glial staining in other areas of the brain. **P < 0.005 compared to sham mice. See also Table 2. Representative photomicrographs of IBA1 **(E)**, CD45 **(F)**, and GFAP **(G)** in the focus region of the neocortex show the robust glial morphological activation that occurs in response to the injury.

To determine what effect, if any, MW151 had on microglia and astrocyte morphological activation, we measured IBA1, CD45, and GFAP immunohistochemistry. Using the Aperio ScanScope digital slide scanner and image software, we identified five brain regions of interest (ROI), which were outlined as illustrated in Figure 4A. Quantification of the digital slide was done using the positive pixel algorithm for the five ROIs. As shown by the heatmap in Figure 4B,C,D, the largest increase in glial activation in the CHI mice compared to the sham mice was in the neocortex most proximal to the point of impact. The focus region of the neocortex, defined by an area that extended laterally from midline by 1.75 cm, showed a large increase in staining for all three markers (Figure 4E,F,G). In addition to the focus region, there were injury-induced increases in staining in the entire neocortex (which included the focus regions), in the entorhinal cortex, and in the corpus callosum, but not in the hippocampus (see Table 2). Treatment with MW151 in the extended treatment paradigm (3 h, 6 h, and 1 to 7 days p.i.) reduced IBA1, CD45, and GFAP staining in the focus region of the cortex. There was little effect of MW151 treatment on glial staining in other areas of the brain. These IHC results cannot be directly compared to our prior study [16], where a different MW151 dosing paradigm and experimental protocols were used. However, the overall finding that repeat dosing with MW151 during the pathophysiology progression time window can alter glia IHC is consistent across studies.

**Table 2:** **Effect of CHI and MW151 treatment on IBA1, CD45, and GFAP staining**

**IHC stain**	**Brain region**	**Sham + veh**	**CHI + veh**	**CHI + MW151**
		**(** ***n*** **= 11)**	**(** ***n*** **= 14)**	**(** ***n*** **= 14)**
IBA1	Neocortex: focus	0.0 ± 0.2	0.3 ± 0.5	0.2 ± 0.3
	Neocortex: all	0.0 ± 0.1	0.1 ± 0.2	0.0 ± 0.2
	Hippocampus	0.0 ± 0.2	0 ± 0.2	−0.1 ± 0.1
	Corpus callosum	0.0 ± 0.5	0.1 ± 0.5	0.1 ± 0.5
	Entorhinal cortex	0.0 ± 0.2	0 ± 0.2	0.0 ± 0.2
CD45	Neocortex: focus	0.0 ± 1.3	7.7 ± 10.4	4.4 ± 5.2
	Neocortex: all	0.0 ± 1.2	2.3 ± 3.6	1.4 ± 2.5
	Hippocampus	0.0 ± 0.7	0.2 ± 0.9	0.3 ± 1.1
	Corpus callosum	0.0 ± 0.6	1.2 ± 1.1	1.3 ± 1.3
	Entorhinal cortex	0.0 ± 0.7	0.6 ± 1.0	0.9 ± 1.5
GFAP	Neocortex: focus	0.0 ± 0.3	4.6 ± 3.7	3.9 ± 3.1
	Neocortex: all	0.0 ± 0.2	1.7 ± 1.4	1.5 ± 1.3
	Hippocampus	0.0 ± 0.1	0.0 ± 0.1	0.0 ± 0.1
	Corpus callosum	0.0 ± 0.2	0.3 ± 0.3	0.4 ± 0.3
	Entorhinal cortex	0.0 ± 0.2	0.4 ± 0.3	0.4 ± 0.3

Data expressed as change from sham + veh baseline (mean ± SD). CHI, closed head injury; IHC, immunohistochemical; GFAP, glial fibrillary acidic protein; veh, vehicle.

## Conclusions

Our goal is to develop safe and effective therapeutics that alter the pathological progression of TBI by reducing the potential for destructive glia inflammation/neuron dysfunction cycles and their long-term cognitive effects. Consistent with this, MW151 has a documented disease-modifying efficacy in diverse animal models in which cognitive dysfunction is brought about by injury- or disease-induced overproduction of proinflammatory cytokines [9-16,26]. The results presented here extend the potential clinical utility of MW151 in CHI by documenting efficacy in a standard preclinical model of diffuse TBI. Exploration of dosing revealed that MW151 exerts its pharmacological mechanism of action at low concentrations even when administered up to 6 h following CHI. In addition, repeat administration of MW151 during the first 7 days after injury reduced the glial activation and cognitive impairment seen at 14 days after injury. Overall, the extended pharmacodynamics effect when MW151 intervention is performed during post-injury proinflammatory cytokine up-regulation is consistent with MW151 pharmacological mechanism of action and indicates the potential for efficacious use of MW151 administration up to several hours after injury. These results suggest that a therapeutic strategy targeting neuroinflammatory responses following TBI, such as with MW151, may have potential to be translated into improved clinical outcomes in human head injury.

## Abbreviations

CHI: Closed head injury
CNS: Central nervous system
i.p.: Intraperitoneal
IL-1: Interleukin-1
MSD: Meso scale discovery
p.i.: Post-injury
TBI: Traumatic brain injury
veh: Vehicle

## References

[1] Faul M, Xu L, Wald MM, Coronado VG. Traumatic brain injury in the United States: emergency department visits, hospitalizations, and deaths. Centers for Disease Control and Prevention, National Center for Injury Prevention and Control Atlanta (GA): 2010.

[2] Bazarian JJ, Cernak I, Noble-Haeusslein L, Potolicchio S, Temkin N (2009). Long-term neurologic outcomes after traumatic brain injury. J Head Trauma Rehabil.

[3] Jordan BD (2014). Chronic traumatic encephalopathy and other long-term sequelae. Continuum (Minneap Minn).

[4] Ramlackhansingh AF, Brooks DJ, Greenwood RJ, Bose SK, Turkheimer FE, Kinnunen KM (2011). Inflammation after trauma: microglial activation and traumatic brain injury. Ann Neurol.

[5] Heneka MT, Kummer MP, Latz E (2014). Innate immune activation in neurodegenerative disease. Nat Rev Immunol.

[6] Mosher KI, Wyss-Coray T (2014). Microglial dysfunction in brain aging and Alzheimer’s disease. Biochem Pharmacol.

[7] Kumar A, Loane DJ (2012). Neuroinflammation after traumatic brain injury: opportunities for therapeutic intervention. Brain Behav Immun.

[8] Woodcock T, Morganti-Kossmann MC (2013). The role of markers of inflammation in traumatic brain injury. Front Neurol.

[9] Hu W, Ralay Ranaivo H, Roy SM, Behanna HA, Wing LK, Munoz L (2007). Development of a novel therapeutic suppressor of brain proinflammatory cytokine up-regulation that attenuates synaptic dysfunction and behavioral deficits. Bioorg Med Chem Lett.

[10] Bachstetter AD, Norris CM, Sompol P, Wilcock DM, Goulding D, Neltner JH (2012). Early stage drug treatment that normalizes proinflammatory cytokine production attenuates synaptic dysfunction in a mouse model that exhibits age-dependent progression of Alzheimer’s disease-related pathology. J Neurosci.

[11] Somera-Molina KC, Robin B, Somera CA, Anderson C, Stine C, Koh S (2007). Glial activation links early-life seizures and long-term neurologic dysfunction: evidence using a small molecule inhibitor of proinflammatory cytokine upregulation. Epilepsia.

[12] Somera-Molina KC, Nair S, Van Eldik LJ, Watterson DM, Wainwright MS (2009). Enhanced microglial activation and proinflammatory cytokine upregulation are linked to increased susceptibility to seizures and neurologic injury in a ‘two-hit’ seizure model. Brain Research.

[13] Karpus WJ, Reynolds N, Behanna HA, Van Eldik LJ, Watterson DM (2008). Inhibition of experimental autoimmune encephalomyelitis by a novel small molecular weight proinflammatory cytokine suppressing drug. J Neuroimmunol.

[14] Chrzaszcz M, Venkatesan C, Dragisic T, Watterson DM, Wainwright MS (2010). Minozac treatment prevents increased seizure susceptibility in a mouse ‘two-hit’ model of closed skull traumatic brain injury and electroconvulsive shock-induced seizures. J Neurotrauma.

[15] Jenrow KA, Brown SL, Lapanowski K, Naei H, Kolozsvary A, Kim JH (2013). Selective inhibition of microglia-mediated neuroinflammation mitigates radiation-induced cognitive impairment. Radiat Res.

[16] Lloyd E, Somera-Molina K, Van Eldik LJ, Watterson DM, Wainwright MS (2008). Suppression of acute proinflammatory cytokine and chemokine upregulation by post-injury administration of a novel small molecule improves long-term neurologic outcome in a mouse model of traumatic brain injury. J Neuroinflammation.

[17] Landis SC, Amara SG, Asadullah K, Austin CP, Blumenstein R, Bradley EW (2012). A call for transparent reporting to optimize the predictive value of preclinical research. Nature.

[18] Shineman DW, Basi GS, Bizon JL, Colton CA, Greenberg BD, Hollister BA (2011). Accelerating drug discovery for Alzheimer’s disease: best practices for preclinical animal studies. Alzheimers Res Ther.

[19] Brody DL, Mac Donald C, Kessens CC, Yuede C, Parsadanian M, Spinner M (2007). Electromagnetic controlled cortical impact device for precise, graded experimental traumatic brain injury. J Neurotrauma.

[20] Webster SJ, Bachstetter AD, Van Eldik LJ (2013). Comprehensive behavioral characterization of an APP/PS-1 double knock-in mouse model of Alzheimer’s disease. Alzheimers Res Ther.

[21] Bachstetter AD, Rowe RK, Kaneko M, Goulding D, Lifshitz J, Van Eldik LJ (2013). The p38alpha MAPK regulates microglial responsiveness to diffuse traumatic brain injury. J Neurosci.

[22] Xiong Y, Mahmood A, Chopp M (2013). Animal models of traumatic brain injury. Nat Rev Neurosci.

[23] Craft JM, Watterson DM, Frautschy SA, Van Eldik LJ (2004). Aminopyridazines inhibit beta-amyloid-induced glial activation and neuronal damage in vivo. Neurobiol Aging.

[24] Craft JM, Van Eldik LJ, Zasadzki M, Hu W, Watterson DM (2004). Aminopyridazines attenuate hippocampus-dependent behavioral deficits induced by human beta-amyloid in a murine model of neuroinflammation. J Mol Neurosci.

[25] Craft JM, Watterson DM, Van Eldik LJ (2006). Human amyloid beta-induced neuroinflammation is an early event in neurodegeneration. Glia.

[26] Macauley SL, Wong AM, Shyng C, Augner DP, Dearborn JT, Pearse Y (2014). An anti-neuroinflammatory that targets dysregulated glia enhances the efficacy of CNS-directed gene therapy in murine infantile neuronal ceroid lipofuscinosis. J Neurosci.

[27] James ML, Wang H, Cantillana V, Lei B, Kernagis DN, Dawson HN (2012). TT-301 inhibits microglial activation and improves outcome after central nervous system injury in adult mice. Anesthesiology.

